# Association Between Common Variants of *APOE*, *ABCA7*, *A2M*, *BACE1*, and Cerebrospinal Fluid Biomarkers in Alzheimer’s Disease: Data from the PUMCH Dementia Cohort

**DOI:** 10.3233/JAD-215067

**Published:** 2022-02-15

**Authors:** Liling Dong, Chenhui Mao, Caiyan Liu, Jie Li, Xinying Huang, Jie Wang, Dan Lei, Shanshan Chu, Longze Sha, Qi Xu, Bin Peng, Liying Cui, Jing Gao

**Affiliations:** a Neurology Department, State Key Laboratory of Complex Severe and Rare Diseases, Peking Union Medical College Hospital, Chinese Academy of Medical Sciences and Peking Union Medical College, Beijing, China; b Institute of Basic Medical Sciences, Peking Union Medical College, Beijing, China

**Keywords:** *ABCA7*, Alzheimer’s disease, amyloid-beta, *APOE*, cerebrospinal fluid, phosphorylated tau, single nucleotide polymorphism

## Abstract

**Background:**

The previous studies have identified several genes in relation to Alzheimer’s disease (AD), such as *ABCA7*, *CR1*, etc. A few studies have explored the association between the common variants, mainly in the non-coding regions of these genes, and cerebrospinal fluid (CSF) biomarkers. Fewer studies target the variants in the coding regions.

**Objective:**

To illustrate the association between the common variants within or adjacent to the coding regions of AD susceptible genes and CSF biomarkers in AD patients.

**Methods:**

75 sporadic probable AD patients were extracted from the dementia cohort of Peking Union Medical College Hospital. They all had history inquiry, physical examination, blood test, cognitive assessment, brain MRI, CSF testing of Aβ_42_, ^181^p-tau, and t-tau, and next-generation DNA sequencing. Sixty-nine common single nucleotide polymorphisms (SNPs) (minor allele frequency > 0.01) within or near the coding region of 13 AD susceptible genes were included in the analysis.

**Results:**

The rs7412-CC (*APOE*) genotype showed lower CSF Aβ_42_ level and higher p-tau/Aβ_42_ ratio than the rs7412-CT genotype. The rs3752246-C (*ABCA7*) allele correlated with lower CSF Aβ_42_ level. The alternate alleles of six *ABCA7* SNPs were related to lower CSF p-tau, including rs3745842, rs3764648, rs3764652, rs4147930, rs4147934 and rs881768. The rs11609582-TT (*A2M*) genotype showed higher CSF p-tau than the rs11609582-TA genotype. The p-tau/Aβ_42_ ratio was higher in the rs490460-TT (*BACE1*) genotype relative to the rs490460-GT genotype.

**Conclusion:**

Some common variants within or near the coding regions of *APOE*, *ABCA7*, *A2M*, and *BACE1* are associated with CSF Aβ_42_, p-tau. or p-tau/Aβ_42_.

## INTRODUCTION

Alzheimer’s disease (AD) is the most prevalent neurodegenerative dementia. In 2010, there were about 3.71 million people in China who lived with AD [1]. However, the genetic basis of AD remains unclear. Pathogenic mutations in the amyloid precursor protein (*APP*), the presenilin 1 (*PSEN1*), and the presenilin 2 (*PSEN2*) genes can explain less than 1% of the AD population [2]. The apolipoprotein E (*APOE*) *ɛ*4 haplotype is a well-known genetic risk factor for AD. It is composed of two single nucleotide polymorphisms (SNPs), rs429358-C and rs7412-C. However, more than half of AD patients do not carry the *APOE* *ɛ*4 allele [3]. Thereby, many studies are devoted to exploring additional susceptibility loci associated with AD.

AD is pathologically characterized by extracellular deposition of amyloid-β (Aβ) plaques and intracellular aggregation of highly phosphorylated tau (p-tau)-containing neurofibrillary tangles [4]. Based on the 2018 AD research framework from the National Institute on Aging-Alzheimer’s Association workgroups, it is Aβ plaques and neurofibrillary tau deposits that define AD as a unique neurodegenerative disease. And low Aβ_42_ and elevated p-tau in cerebrospinal fluid (CSF) are biomarkers of Aβ plaques and fibrillar tau, respectively [5]. In this case, we aim to assist in the exploration of AD risk loci by identifying the risk SNPs which correlate with CSF biomarkers.

Genome-wide association studies and meta-analyses have identified several genes associated with AD risk, such as the alpha 2 Macroglobulin (*A2M*), the ATP-Binding Cassette Subfamily A Member 7 (*ABCA7*), the beta-Site amyloid precursor protein-cleaving enzyme 1 (*BACE1*), the bridging integrator 1 (*BIN1*), the Clusterin (*CLU*), the complement component (3b/4b) receptor 1 (*CR1*), the membrane spanning 4-domains A6A (*MS4A6A*), the membrane spanning 4-domains A6E (*MS4A6E*), the NME/NM23 family member 8 (*NME8*), the phosphatidylinositol binding clathrin assembly protein (*PICALM*), the plasminogen activator urokinase (*PLAU*), and the sortilin related receptor 1 (*SORL1*) genes [6–10]. They are involved in cholesterol metabolism, immune response, endocytosis, Aβ processing, etc. [11]. Most susceptibility loci are common variants, with minor allele frequency > 1%.

A few studies have been devoted to exploring the association between susceptibility loci and CSF biomarkers. With the Alzheimer’s Disease Neuroimaging Initiative (ADNI) database, previous studies have investigated the correlation between 15 *ABCA7* and 83 *CR1* SNPs and CSF biomarkers. However, most of the SNPs are in the non-coding regions, such as intron and untranslated region [12, 13]. Fewer studies target the variants located in the coding regions.

We hypothesized that the common SNPs within or near the coding regions of AD risk genes were potential functional loci for AD. The mutations close to the splicing junction might affect mRNA splicing. Missense mutations in the coding region could alter amino acid products. And synonymous mutations might influence promoter activity, pre-mRNAs conformation, stability, protein folding, or function [14–16]. The altered gene products might further affect cholesterol metabolism, immune response, Aβ processing, tau phosphorylation, etc., and ultimately contribute to AD pathogenesis.

In present study, we focused on the common SNPs within or near the coding regions of 13 known AD susceptible genes (*APOE*, *A2M*, *ABCA7*, *BACE1*, *BIN1*, *CLU*, *CR1*, *MS4A6A*, *MS4A6E*, *NME8*, *PICALM*, *PLAU*, and *SORL1*). We would illustrate the association between these SNPs and CSF Aβ_42_, p-tau, and total tau (t-tau).

## METHODS

### Participants

The participants were from the dementia cohort of Peking Union Medical College Hospital. All subjects had basic tests, including history inquiry, physical examination, blood biochemical test, cognitive assessment, and brain CT/MRI. Cognitive assessment included the Mini-Mental State Exam (MMSE), activities of daily living (ADL), etc. 315 cases received whole exon sequencing, and 1,144 cases had targeted exon sequencing of 278 dementia-related genes. CSF testing of Aβ_42_, ^181^p-tau, and t-tau was performed in 362 subjects. Pathological evidence was not obtained.

75 Chinese Han patients were included in the study. The inclusion criteria were as follows: 1) met the diagnostic criteria for probable AD based on 2011 diagnostic guidelines for AD from National Institute on Aging-Alzheimer’s Association workgroups [17]; 2) no family history of dementia, and no potential pathogenic variant implicated in dementia; (3) no missing data on basic tests, gene sequencing, CSF testing of Aβ_42_, ^181^p-tau, and t-tau; and 4) informed consent was obtained. This study was approved by ethics committee of PUMCH (No. JS-1836).

### Gene sequencing

IDT xGen Lockdown Probes were used in whole exon sequencing with 50 bp flanking intron length. The targeted exon sequencing covered 3927 exons with 20 bp flanking intron regions. The DNA libraries were sequenced on Illumina HiSeq X Ten Analyzers (Illumina, San Diego, USA). “Clean reads” were generated with AfterQC [18]. All reads were aligned to human reference genome 19 with Burrows-Wheeler Aligner (v.0.5.9) [19]. Local realignment and base quality recalibration were finished with GATK IndelRealigner and BaseRecalibrator (v3.5). SNVs and small indels were called with GATK UnifiedGenotyper (v 3.5).

This report focused on 13 known AD susceptible genes, including *APOE*, *A2M*, *ABCA7*, *BACE1*, *BIN1*, *CLU*, *CR1*, *MS4A6A*, *MS4A6E*, *NME8*, *PICALM*, *PLAU*, and *SORL1*. Finally, 69 SNPs were included in the analysis. The inclusion criteria were as follows: 1) minor allele frequency > 0.01, according to ExAC, Genomes databases, etc.; 2) located in the exon region or within 10 bp of splicing junction.

### CSF testing

CSF samples was collected using low protein binding tube (Eppendorf Protein LoBind Tube; 1.5 ml; Hamburg, Germany). The tubes were filled more than 75%. Within one day, the samples were centrifuged at 1800 rpm, 4°C for 10 min. After that, the supernatant was moved to new tubes and stored in freezers at –80°C. Within two weeks, CSF Aβ_42_, ^181^p-tau, and t-tau were measured using ELISA method (INNOTEST hTAU Ag, PHOSPHO-TAU, β-AMYLOID (1–42); Fujirebio, Ghent, Belgium).

The CSF biomarker cut-off values for clinical AD in our laboratory were submitted to Alzheimer’s Association International Conference in 2019 (https://doi.org/10.1016/j.jalz.2019.06.790). With the increasing sample size, the current CSF biomarker cut-offs for clinical AD have been set as follows: Aβ_42_ <570.9 pg/ml, p-tau > 56.49 pg/ml, t-tau > 241.6 pg/ml, p-tau/Aβ_42_ > 0.08465, and t-tau/Aβ_42_ > 0.5290. The sensitivities of these cut-offs are 56.6%, 88.7%, 81.1%, 77.4%, and 84.9%, respectively. The specificities are 84.6%, 84.6%, 90.4%, 90.4%, and 90.4%, respectively. These cut-offs are unpublished data.

### Statistical analysis

**Fig. 1:**
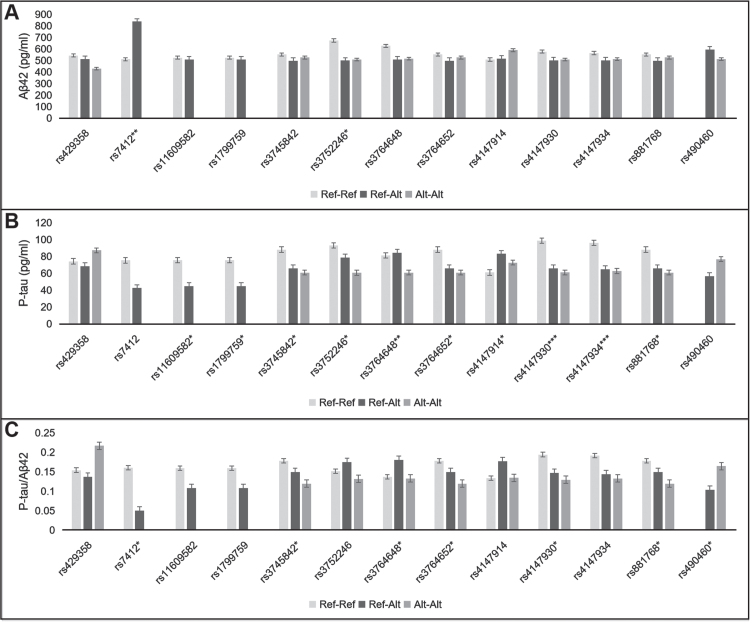
Effect of 13 SNPs on CSF Aβ_42_ (A), p-tau (B), and p-tau/Aβ_42_ ratio (C). Ref-Ref, reference allele homozygote; Ref-Alt, heterozygote; Alt-Alt, alternate allele homozygote. ^*^p < 0.05, ^**^p < 0.01, ^***^p < 0.001.

The CSF biomarker data (Aβ_42_, ^181^p-tau, t-tau, ^181^p-tau/Aβ_42_, t-tau/Aβ_42_) were compared by analysis of covariate (ANCOVA). Sex, age, disease course, and *APOE* status (*ɛ*4 carrier or non-carrier) were included in the model as fixed factor or covariate. Unadjusted means were displayed in Fig. 1 and Supplementary Tables 2 and 3.

## RESULTS

### Demographic characteristics

As shown in Table 1, there were 75 subjects: 35 male and 40 female. They had an average age of 61.7±7.7 years old. The average disease course was 3.5±2.8 years. None of them had positive family history of dementia. None of them harbored potential pathogenic mutations implicated in dementia.

**Table 1:** General characteristics of 75 subjects

Demographics
Male	35 (46.7%)
Female	40 (53.3%)
Age (y)	61.7±7.7
Disease course (y)	3.5±2.8
*APOE* genotype
*ɛ*2*ɛ*3	3 (4.0%)
*ɛ*3*ɛ*3	44 (58.7%)
*ɛ*3*ɛ*4	20 (26.7%)
*ɛ*4*ɛ*4	8 (10.7%)
*ɛ*2 allele frequency	2.0%
*ɛ*3 allele frequency	74.0%
*ɛ*4 allele frequency	24.0%
Cognitive function
MMSE	14.0±8.3
ADL	38.3±11.2
CSF biomarker
Aβ_42_ (pg/ml)	523.0±175.0
P-tau (pg/ml)	74.0±31.8
T-tau (pg/ml)	688.7±734.7
P-tau/Aβ_42_	0.16±0.08
T-tau/Aβ_42_	1.38±1.26
Aβ_42_ <570.9 pg/ml /≥570.9 pg/ml	53 (70.7%) / 22 (29.3%)
p-tau > 56.49 pg/ml /≤56.49 pg/ml	56 (74.7%) / 19 (25.3%)
t-tau > 241.6 pg/ml /≤241.6 pg/ml	64 (85.3%) / 11 (14.7%)
p-tau/Aβ_42_ > 0.08465 /≤0.08465	63 (84.0%) / 12 (16.0%)
t-tau/Aβ_42_ > 0.5290 /≤0.5290	59 (78.7%) / 16 (21.3%)

### SNPs frequency

As shown in Supplementary Table 1, there were 69 SNPs in 13 risk genes. Of them, 58 were in the exon (24 missense and 34 synonymous), whereas 11 were within 10 bp of splicing junction in the intron.

Compared with the allele frequency in east Asian populations from 1000 Genomes database, the cohort had higher rs429358-C (*APOE*) (24.0% versus 8.6%) and lower rs7412-T (*APOE*) allele frequencies (2.0% versus 10.0%). Additionally, the cohort showed lower rs2274567-G (*CR1*) (15.3% versus 32.7%), rs3811381-G (*CR1*) (15.3% versus 32.2%), rs6691117-G (*CR1*) (17.3% versus 34.3%), and higher rs12364988-C (*SORL1*) (46.7% versus 31.2%) allele frequencies. As for the other SNPs, the alternate allele frequencies in this AD cohort were comparable to those from 1000 Genomes database.

### Association between SNPs and CSF biomarkers

As illustrated in Fig. 1 and Supplementary Tables 2 and 3, 13 SNPs were relevant to CSF biomarkers, including two *APOE*, two *A2M*, one *BACE1*, and eight *ABCA7* SNPs. They were associated with CSF Aβ_42_, p-tau or p-tau/Aβ_42_. None of the 69 SNPs had effects on CSF t-tau or t-tau/Aβ_42_.

After adjustment by the rs429358 (*APOE*), the rs7412-CC (*APOE*) genotype showed lower Aβ_42_ level and higher p-tau/Aβ_42_ ratio than the rs7412-CT genotype (509.9±162.7 versus 836.7±197.9 pg/ml, *p* = 0.006; 0.16±0.08 versus 0.05±0.00, *p* =0.022). Among the rs429358-TT, rs429358-TC, and rs429358-CC genotypes, CSF Aβ_42_ level showed a declining trend (543.3±191.9, 513.0±141.2, and 428.2±120.1 pg/ml, *p* = 0.363). The rs429358-CC genotype showed higher p-tau/Aβ_42_ ratio than the rs429358-TT and rs429358-TC genotypes (0.22±0.07 versus 0.15±0.09, 0.14±0.05, *p* = 0.050). However, some of these differences did not reach statistical significance.

The rs3752246-C (*ABCA7*) allele correlated with lower Aβ_42_ level (CC, GC versus GG: 508.3±179.4, 498.7±150.7 versus 672.4±205.1 pg/ml, *p* =0.047). The alternate alleles of seven *ABCA7* SNPs were related to lower p-tau, including rs3745842, rs3752246, rs3764648, rs3764652, rs4147930, rs4147934, and rs881768. Of them, the alternate alleles of rs3745842, rs3764652, rs4147930, and rs881768 were also relevant to lower p-tau/Aβ_42_ ratio. In addition, the rs4147914-GA genotype showed higher p-tau level than the rs4147914-AA and rs4147914-GG genotypes (83.0±31.8 versus 72.6±44.0, 60.9±21.6 pg/ml, *p* = 0.018).

The rs11609582-TT (*A2M*) genotype showed higher p-tau level than the rs11609582-TA genotype (75.6±31.8 versus 44.7±16.6 pg/ml, *p* = 0.029). Also, the reference allele homozygote of rs1799759 had higher p-tau than the rs1799759 heterozygote (75.6±31.8 versus 44.7±16.6 pg/ml, *p* = 0.029).

The rs490460-TT (*BACE1*) genotype showed lower Aβ_42_ and higher p-tau levels than the rs490460-GT genotype (512.1±159.7 versus 593.5±253.0pg/ml, *p* = 0.58; 76.7±32.4 versus 56.7±21.9 pg/ml, *p* = 0.12). These differences were not statistically significant. However, the p-tau/Aβ_42_ ratio was significantly higher in the rs490460-TT genotype relative to the rs490460-GT genotype (0.16±0.08 versus 0.10±0.04, *p* = 0.04).

### Sex^*^SNP interaction

As shown in Table 2, two SNPs showed sex^*^SNPs interactions on CSF Aβ_42_ level, including the rs3752240 (*ABCA7*) (*F* = 4.371, *p* = 0.016) and the rs7232 (*MS4A6A*) (*F* = 6.696, *p* = 0.012). Two *NME8* SNPs showed sex^*^SNPs interactions on p-tau/Aβ_42_, including the rs2598044 (*F* = 4.811, *p* = 0.011) and the rs2722372 (*F* = 4.811, *p* = 0.011).

**Table 2:** Sex^*^SNP interactions on CSF biomarkers [F value (*p* value)]

Main effect	Aβ_42_	P-tau/Aβ_42_
Sex	3.324 (0.073)
rs3752240 (*ABCA7*)	5.347 (0.007)
Sex^*^rs3752240	4.371 (0.016)
Sex	9.458 (0.003)	6.135 (0.016)
rs7232 (*MS4A6A*)	1.269 (0.264)	0.532 (0.468)
Sex^*^rs7232	6.696 (0.012)	6.422 (0.014)
Sex		1.834 (0.180)
rs2598044 (*NME8*)		0.509 (0.604)
Sex^*^rs2598044		4.811 (0.011)
Sex		1.834 (0.180)
rs2722372 (*NME8*)		0.509 (0.604)
Sex^*^rs2722372		4.811 (0.011)

Among the subjects with the rs3752240-AA and the rs7232-AT genotypes, males had lower Aβ_42_ level than females. Among the participants with the rs2598044-CT and the rs2722372-GA genotypes, the p-tau/Aβ_42_ ratio was higher in males relative to females. However, among those with rs3752240-AG, rs7232-AA, rs2598044-CC, and rs2722372-GG genotypes, Aβ_42_ or p-tau/Aβ_42_ ratio did not differ between males and females.

## DISCUSSION

### APOE, ABCA7, A2M, BACE1 SNPs and CSF Aβ_42_, p-tau

The *APOE* *ɛ*4 allele is a well-known genetic risk factor for AD. Conversely, the *ɛ*2 allele confers a reduced AD risk [20]. These are further confirmed in this study. The *ɛ*2 and *ɛ*4 allele frequencies in the cohort are 2.0% and 24.0%, respectively, which are lower and higher than those in Chinese general population (10.5%, 7.1%) [21]. Besides, we find the rs7412-T allele is relevant to increased CSF Aβ_42_ level, and the rs429358-C allele shows a trend associated with decreased Aβ_42_. The correlation between these *APOE* SNPs and CSF Aβ_42_ level is related to the role of *APOE* in Aβ metabolism. The *APOE* *ɛ*4 can increase Aβ production by improving the activity of *γ*-secretase and inhibit Aβ clearance by impairing its lysosomal and proteolytic degradation [22, 23]. Comparatively, the involvement of the *APOE* *ɛ*2 in AD is less clear. Compared with the *APOE* *ɛ*4, the *APOE* *ɛ*2 allele is more efficient at promoting the degradation of soluble Aβ and its transport across vessel walls [24].

Among the 13 potential functional SNPs, 61.5% (8/13) SNPs are from the *ABCA7*. Six novel SNPs (rs3764648, rs3764652, rs4147930, rs4147934, rs881768, and rs4147914) show effects on CSF p-tau level. Two known SNPs (rs3752246, rs3745842) correlate with Aβ_42_ and p-tau burden, respectively. Like the *APOE*, the *ABCA7* is also located on chromosome 19q13.3. It has 47 exons. It might influence Aβ production, aggregation by mediating β-secretase cleavage, microglial endocytosis, or macrophage phagocytosis [25]. Aβ deposition can initiate concurrent accumulation of tau tangles [26].

Two novel *A2M* SNPs (rs11609582, rs1799759) are related to CSF p-tau burden. The *A2M* might affect tau pathogenesis by its interaction with calcineurin and regulator of calcineurin 1, which are the enzyme and regulator of tau phosphorylation, respectively [27].

One novel *BACE1* SNP (rs490460) is relevant to p-tau/Aβ_42_ ratio. This might be due to the role of the *BACE1* in Aβ metabolism. The *BACE1* protease is implicated in the β-site cleaving of amyloid precursor protein, which can eventually lead to the synthesis of Aβ peptide [28].

### Allele frequency variation of CR1, SORL1

The alternate allele frequencies of rs2274567 (*CR1*), rs3811381 (*CR1*), rs6691117 (*CR1*), and rs12364988-C (*SORL1*) in this cohort are far from those in the East Asian population from 1000 Genomes database. However, these allele frequency data in this cohort are close to the data from some previous studies. In Chinese late-onset AD patients, the minor allele frequencies of rs2274567, rs3811381, and rs6691117 are 12%, 14%, and 11%, respectively. In Chinese cognitively normal controls, the minor allele frequencies are 19%, 18%, and 19%, respectively [29, 30]. In Italian late-onset AD patients and normal controls, the rs12364988-G allele frequencies are 59.3% and 53.4%, respectively [31]. These allele frequency variations might be attributed to ethnic differences. Further epidemiological investigation is expected.

### Sex^*^SNP (ABCA7, MS4A6A, NME8) interaction

This is the first time that we demonstrate the interactions between sex and *ABCA7*, *MS4A6A*, and *NME8* on CSF biomarkers. Among the subjects with rs3752240-AA (*ABCA7*), rs7232-AT (*MS4A6A*), rs2598044-CT (*NME8*), and rs2722372-GA (*NME8*) genotypes, males have lower Aβ_42_ or higher p-tau/Aβ_42_ than females. The sex differences in the effect of these SNPs on CSF biomarkers might be somewhat responsible for the sex differences in the amyloid load. Cavedo et al. has found that there is higher anterior cingulate cortex amyloid burden in men relative to women [32]. However, previous research on sex differences in AD shows that women have more tau pathology than men [33].

### P-tau biased association

In this paper, ten SNPs show effects on CSF p-tau. Comparatively, there is less association between these SNPs and CSF Aβ_42_. This might be partly due to the dynamic evolution of CSF biomarkers at different stages of cognitive impairment. The CSF Aβ_42_ level decreases mainly in the preclinical stage and reaches a plateau in the dementia stage, whereas the CSF tau level increases gradually in the mild dementia stage [34–36]. T-tau is a non-specific biomarker for AD which reflects the intensity of neuronal injury, whereas p-tau is relatively specific for AD which indicates a pathological state of paired helical filament tau deposits [5]. This might explain why these SNPs are associated with p-tau rather than t-tau.

### Conclusion and limitation

This study involves 69 common SNPs within or near coding regions of 13 AD risk genes. *APOE*, *ABCA7*, *A2M*, and *BACE1* SNPs show associations with CSF Aβ_42_, p-tau, and p-tau/Aβ_42_, including four known and nine novel SNPs. *ABCA7*, *MS4A6A*, and *NME8* have sex^*^SNPs interactions on CSF Aβ_42_ and p-tau/Aβ_42_.

The main limitation of this study is the small sample size and absence of pathological evidence. All the subjects in this study are clinically diagnosed with probable AD. None of them have pathological evidence. According to the CSF biomarker cut-offs for AD in our laboratory, only 50.7% (38/75) subjects reach the cutoff values of all the five variables. 29.3% (22/75) subjects have CSF Aβ_42_ > 570.9 pg/ml, and 25.3% (19/75) subjects have CSF p-tau <56.49 pg/ml. The atypical CSF profile might be attributed to individual differences in CSF dynamics. Grothe studied the CSF biomarkers in pathology-confirmed AD patients from ADNI database. They reported a considerably higher CSF Aβ_42_ cutoff (1,097 pg/ml) for differentiating high and low Thal phases, as well as a lower p-tau cutoff (19 pg/ml) for discriminating high and low neuritic plaque scores [37]. In this case, pathological evidence is highly expected in order to reduce the bias of the study. Next, we expect to expand the sample size and strive for pathological evidence. We will explore the synergistic effect of multiple SNPs on CSF biomarkers. As for the potential susceptibility loci, functional analysis should be considered.

## Supplementary Material

Supplementary Material

## References

[1] Chan KY , Wang W , Wu JJ , Liu L , Theodoratou E , Car J , Middleton L , Russ TC , Deary IJ , Campbell H , Wang W , Rudan I (2013) Epidemiology of Alzheimer’s disease and other forms of dementia in China, 1990-2010: A systematic review and analysis. Lancet 381, 2016–2023. 23746902 10.1016/S0140-6736(13)60221-4

[2] Cacace R , Sleegers K , Van Broeckhoven C (2016) Molecular genetics of early-onset Alzheimer’s disease revisited. Alzheimers Dement 12, 733–748. 27016693 10.1016/j.jalz.2016.01.012

[3] Ward A , Crean S , Mercaldi CJ , Collins JM , Boyd D , Cook MN , Arrighi HM (2012) Prevalence of apolipoprotein E4 genotype and homozygotes (APOE e4/4) among patients diagnosed with Alzheimer’s disease: A systematic review and meta-analysis. Neuroepidemiology 38, 1–17. 22179327 10.1159/000334607

[4] Scheltens P , Blennow K , Breteler MM , de Strooper B , Frisoni GB , Salloway S , Van der Flier WM (2016) Alzheimer’s disease. Lancet 388, 505–517. 26921134 10.1016/S0140-6736(15)01124-1

[5] Jack CJ , Bennett DA , Blennow K , Carrillo MC , Dunn B , Haeberlein SB , Holtzman DM , Jagust W , Jessen F , Karlawish J , Liu E , Molinuevo JL , Montine T , Phelps C , Rankin KP , Rowe CC , Scheltens P , Siemers E , Snyder HM , Sperling R (2018) NIA-AA Research Framework: Toward a biological definition of Alzheimer’s disease. Alzheimers Dement 14, 535–562. 29653606 10.1016/j.jalz.2018.02.018 PMC5958625

[6] Hollingworth P , Harold D , Sims R , Gerrish A , Lambert JC , Carrasquillo MM , Abraham R , Hamshere ML , Pahwa JS , Moskvina V , et al. (2011) Common variants at ABCA7, MS4A6A/MS4A4E, EPHA1, CD33 and CD2AP are associated with Alzheimer’s disease. Nat Genet 43, 429–435. 21460840 10.1038/ng.803 PMC3084173

[7] Lambert JC , Ibrahim-Verbaas CA , Harold D , Naj AC , Sims R , Bellenguez C , DeStafano AL , Bis JC , Beecham GW , Grenier-Boley B , et al. (2013) Meta-analysis of 74,046 individuals identifies 11 new susceptibility loci for Alzheimer’s disease. Nat Genet 45, 1452–1458. 24162737 10.1038/ng.2802 PMC3896259

[8] Damotte V , van der Lee SJ , Chouraki V , Grenier-Boley B , Simino J , Adams H , Tosto G , White C , Terzikhan N , Cruchaga C , Knol MJ , Li S , Schraen S , Grove ML , Satizabal C , Amin N , Berr C , Younkin S , Gottesman RF , Buee L , Beiser A , Knopman DS , Uitterlinden A , DeCarli C , Bressler J , DeStefano A , Dartigues JF , Yang Q , Boerwinkle E , Tzourio C , Fornage M , Ikram MA , Amouyel P , de Jager P , Reitz C , Mosley TH , Lambert JC , Seshadri S , van Duijn CM (2021) Plasma amyloid beta levels are driven by genetic variants near APOE, BACE1, APP, PSEN2: A genome-wide association study in over 12,000 non-demented participants. Alzheimers Dement 17, 1663–1674. 34002480 10.1002/alz.12333 PMC8597077

[9] Wu W , Jiang H , Wang M , Zhang D (2013) Meta-analysis of the association between urokinase-plasminogen activator gene rs2227564 polymorphism and Alzheimer’s disease. Am J Alzheimers Dis Other Demen 28, 517–523. 23813610 10.1177/1533317513494450 PMC10852686

[10] Xu X , Wang Y , Wang L , Liao Q , Chang L , Xu L , Huang Y , Ye H , Xu L , Chen C , Shen X , Zhang F , Ye M , Wang Q , Duan S (2013) Meta-analyses of 8 polymorphisms associated with the risk of the Alzheimer’s disease. PLoS One 8, e73129. 24039871 10.1371/journal.pone.0073129 PMC3769354

[11] Giri M , Zhang M , Lu Y (2016) Genes associated with Alzheimer’s disease: An overview and current status. Clin Interv Aging 11, 665–681. 27274215 10.2147/CIA.S105769 PMC4876682

[12] Zhao QF , Wan Y , Wang HF , Sun FR , Hao XK , Tan MS , Tan CC , Zhang DQ , Tan L , Yu JT (2016) ABCA7 genotypes confer Alzheimer’s disease risk by modulating amyloid-beta pathology. J Alzheimers Dis 52, 693–703. 27003212 10.3233/JAD-151005

[13] Zhu XC , Dai WZ , Ma T (2020) Impacts of CR1 genetic variants on cerebrospinal fluid and neuroimaging biomarkers in Alzheimer’s disease. BMC Med Genet 21, 181. 32919460 10.1186/s12881-020-01114-x PMC7488421

[14] Capon F , Allen MH , Ameen M , Burden AD , Tillman D , Barker JN , Trembath RC (2004) A synonymous SNP of the corneodesmosin gene leads to increased mRNA stability and demonstrates association with psoriasis across diverse ethnic groups. Hum Mol Genet 13, 2361–2368. 15333584 10.1093/hmg/ddh273

[15] Kimchi-Sarfaty C , Oh JM , Kim IW , Sauna ZE , Calcagno AM , Ambudkar SV , Gottesman MM (2007) A “silent” polymorphism in the MDR1 gene changes substrate specificity. Science 315, 525–528. 17185560 10.1126/science.1135308

[16] Hunt R , Sauna ZE , Ambudkar SV , Gottesman MM , Kimchi-Sarfaty C (2009) Silent (synonymous) SNPs: Should we care about them? Methods Mol Biol 578, 23–39. 19768585 10.1007/978-1-60327-411-1_2

[17] McKhann GM , Knopman DS , Chertkow H , Hyman BT , Jack CJ , Kawas CH , Klunk WE , Koroshetz WJ , Manly JJ , Mayeux R , Mohs RC , Morris JC , Rossor MN , Scheltens P , Carrillo MC , Thies B , Weintraub S , Phelps CH (2011) The diagnosis of dementia due to Alzheimer’s disease: Recommendations from the National Institute on Aging-Alzheimer’s Association workgroups on diagnostic guidelines for Alzheimer’s disease. Alzheimers Dement 7, 263–269. 21514250 10.1016/j.jalz.2011.03.005 PMC3312024

[18] Chen S , Huang T , Zhou Y , Han Y , Xu M , Gu J (2017) AfterQC: Automatic filtering, trimming, error removing and quality control for fastq data. BMC Bioinformatics 18, 80. 28361673 10.1186/s12859-017-1469-3 PMC5374548

[19] Li H , Durbin R (2010) Fast and accurate long-read alignment with Burrows-Wheeler transform. Bioinformatics 26, 589–595. 20080505 10.1093/bioinformatics/btp698 PMC2828108

[20] Farrer LA , Cupples LA , Haines JL , Hyman B , Kukull WA , Mayeux R , Myers RH , Pericak-Vance MA , Risch N , van Duijn CM (1997) Effects of age, sex, and ethnicity on the association between apolipoprotein E genotype and Alzheimer disease. A meta-analysis. APOE and Alzheimer Disease Meta Analysis Consortium. JAMA 278, 1349–1356. 9343467

[21] Giau VV , Bagyinszky E , An SS , Kim SY (2015) Role of apolipoprotein E in neurodegenerative diseases. Neuropsychiatr Dis Treat 11, 1723–1737. 26213471 10.2147/NDT.S84266 PMC4509527

[22] Lane-Donovan C , Herz J (2017) The ApoE receptors Vldlr and Apoer2 in central nervous system function and disease. J Lipid Res 58, 1036–1043. 28292942 10.1194/jlr.R075507 PMC5454520

[23] Jiang Q , Lee CY , Mandrekar S , Wilkinson B , Cramer P , Zelcer N , Mann K , Lamb B , Willson TM , Collins JL , Richardson JC , Smith JD , Comery TA , Riddell D , Holtzman DM , Tontonoz P , Landreth GE (2008) ApoE promotes the proteolytic degradation of Abeta. Neuron 58, 681–693. 18549781 10.1016/j.neuron.2008.04.010 PMC2493297

[24] Yamazaki Y , Zhao N , Caulfield TR , Liu CC , Bu G (2019) Apolipoprotein E and Alzheimer disease: Pathobiology and targeting strategies. Nat Rev Neurol 15, 501–518. 31367008 10.1038/s41582-019-0228-7 PMC7055192

[25] Aikawa T , Holm ML , Kanekiyo T (2018) ABCA7 and pathogenic pathways of Alzheimer’s disease. Brain Sci 8, 27–29. 10.3390/brainsci8020027 PMC5836046 29401741

[26] D’Errico P , Meyer-Luehmann M (2020) Mechanisms of pathogenic tau and abeta protein spreading in Alzheimer’s disease. Front Aging Neurosci 12, 265. 33061903 10.3389/fnagi.2020.00265 PMC7481386

[27] Varma VR , Varma S , An Y , Hohman TJ , Seddighi S , Casanova R , Beri A , Dammer EB , Seyfried NT , Pletnikova O , Moghekar A , Wilson MR , Lah JJ , O’Brien RJ , Levey AI , Troncoso JC , Albert MS , Thambisetty M (2017) Alpha-2 macroglobulin in Alzheimer’s disease: A marker of neuronal injury through the RCAN1 pathway. Mol Psychiatry 22, 13–23. 27872486 10.1038/mp.2016.206 PMC5726508

[28] Vassar R , Kuhn PH , Haass C , Kennedy ME , Rajendran L , Wong PC , Lichtenthaler SF (2014) Function, therapeutic potential and cell biology of BACE proteases: Current status and future prospects. J Neurochem 130, 4–28. 24646365 10.1111/jnc.12715 PMC4086641

[29] Ma XY , Yu JT , Tan MS , Sun FR , Miao D , Tan L (2014) Missense variants in CR1 are associated with increased risk of Alzheimer’ disease in Han Chinese. Neurobiol Aging 35, 417–443. 10.1016/j.neurobiolaging.2013.08.009 24018213

[30] Long Z , Du Y , Li H , Han B (2018) CR1 gene polymorphisms in Chinese patients with paroxysmal nocturnal hemoglobinuria. Gene 659, 149–154. 29551496 10.1016/j.gene.2018.03.037

[31] Cellini E , Tedde A , Bagnoli S , Pradella S , Piacentini S , Sorbi S , Nacmias B (2009) Implication of sex and SORL1 variants in Italian patients with Alzheimer disease. Arch Neurol 66, 1260–1266. 19822782 10.1001/archneurol.2009.101

[32] Cavedo E , Chiesa PA , Houot M , Ferretti MT , Grothe MJ , Teipel SJ , Lista S , Habert MO , Potier MC , Dubois B , Hampel H (2018) Sex differences in functional and molecular neuroimaging biomarkers of Alzheimer’s disease in cognitively normal older adults with subjective memory complaints. Alzheimers Dement 14, 1204–1215. 30201102 10.1016/j.jalz.2018.05.014

[33] Hohman TJ , Dumitrescu L , Barnes LL , Thambisetty M , Beecham G , Kunkle B , Gifford KA , Bush WS , Chibnik LB , Mukherjee S , De Jager PL , Kukull W , Crane PK , Resnick SM , Keene CD , Montine TJ , Schellenberg GD , Haines JL , Zetterberg H , Blennow K , Larson EB , Johnson SC , Albert M , Bennett DA , Schneider JA , Jefferson AL (2018) Sex-specific association of apolipoprotein E with cerebrospinal fluid levels of tau. JAMA Neurol 75, 989–998. 29801024 10.1001/jamaneurol.2018.0821 PMC6142927

[34] Buchhave P , Minthon L , Zetterberg H , Wallin AK , Blennow K , Hansson O (2012) Cerebrospinal fluid levels of beta-amyloid 1-42, but not of tau, are fully changed already 5 to 10 years before the onset of Alzheimer dementia. Arch Gen Psychiatry 69, 98–106. 22213792 10.1001/archgenpsychiatry.2011.155

[35] Jack CJ , Wiste HJ , Vemuri P , Weigand SD , Senjem ML , Zeng G , Bernstein MA , Gunter JL , Pankratz VS , Aisen PS , Weiner MW , Petersen RC , Shaw LM , Trojanowski JQ , Knopman DS (2010) Brain beta-amyloid measures and magnetic resonance imaging atrophy both predict time-to-progression from mild cognitive impairment to Alzheimer’s disease. Brain 133, 3336–3348. 20935035 10.1093/brain/awq277 PMC2965425

[36] Vemuri P , Wiste HJ , Weigand SD , Shaw LM , Trojanowski JQ , Weiner MW , Knopman DS , Petersen RC , Jack CJ (2009) MRI and CSF biomarkers in normal, MCI, and AD subjects: Predicting future clinical change. Neurology 73, 294–301. 19636049 10.1212/WNL.0b013e3181af79fb PMC2715214

[37] Grothe MJ , Moscoso A , Ashton NJ , Karikari TK , Lantero-Rodriguez J , Snellman A , Zetterberg H , Blennow K , Scholl M (2021) Associations of fully automated CSF and novel plasma biomarkers with Alzheimer disease neuropathology at autopsy. Neurology 97, e1229–e1242. 10.1212/WNL.0000000000012513 PMC8480485 34266917

