# Supplementary material for: Association Between Common Variants of APOE, ABCA7, A2M, BACE1, and Cerebrospinal Fluid Biomarkers in Alzheimer’s Disease: Data from the PUMCH Dementia Cohort
Source: J Alzheimers Dis. 2022 Feb 15;85(4):1511–8. doi: 10.3233/JAD-215067 (PMC8925115; doi:10.3233/JAD-215067)
Supplement: Supplementary Material [file jad-85-jad215067-s001.pdf]

Supplementary Material

Association Between Common Variants of *APOE*, *ABCA7*, *A2M*, *BACE1*, and Cerebrospinal Fluid Biomarkers in Alzheimer's Disease: Data from the PUMCH Dementia Cohort

Supplementary Table 1. 69 SNPs and alternate allele frequency

Gene	SNP	Variant	Genotype	Cases (n)	Frequency*	Frequency#
APOE	rs429358	(19q13|NM_000041.3) c.388T>C, p.C130R	TT/TC/CC	47/20/8	24.00%	8.6%
APOE	rs7412	(19q13|NM_000041.3) c.526C>T, p.R176C	CC/CT/TT	72/3/0	2.00%	10.0%
A2M	rs11609582	(12p13|NM_000014) exon21:c.2597-4T>A	TT/TA/AA	71/4/0	2.67%	4.56%
A2M	rs1799759	(12p13|NM_000014.6) c.2126-6_2126-2del	-/HET/HOM	71/4/0	2.67%	4.60%
A2M	rs669	(12p13|NM_000014.4) c.2998A>G, p.I1000V	TT/TC/CC	63/12/0	8.00%	8.20%
ABCA7	rs3745842	(19p13|NM_019112.3) c.4046G>A, p.R1349Q	GG/GA/AA	30/34/11	37.33%	34.80%
ABCA7	rs3752232	(19p13|NM_019112.3) c.955A>G, p.T319A	AA/AG/GG	64/11/0	7.33%	9.20%
ABCA7	rs3752233	(19p13|NM_019112.3) c.1388G>A, p.R463H	GG/GA/AA	58/17/0	11.33%	19.20%
ABCA7	rs3752234	(19p13|NM_019112.3) c.1824A>G, p.A608A	AA/AG/GG	2/26/47	80.00%	75.00%
ABCA7	rs3752237	(19p13|NM_019112.3) c.1851A>G, p.G617G	AA/AG/GG	0/19/56	87.33%	80.60%
ABCA7	rs3752239	(19p13|NM_019112.3) c.2153A>C, p.N718T	AA/AC/CC	60/15/0	10.00%	18.60%
ABCA7	rs3752240	(19p13|NM_019112.3) c.2745A>G, p.V915V	AA/AG/GG	59/13/3	12.67%	11.00%
ABCA7	rs3752241	(19p13|NM_019112.3) c.3417C>G, p.L1139L	CC/CG/GG	56/19/0	12.67%	19.20%
ABCA7	rs3752243	(19p13|NM_019112.3) c.3528A>G, p.L1176L	AA/AG/GG	27/35/13	40.67%	38.20%
ABCA7	rs3752246	(19p13|NM_019112.3) c.4580G>C, p.G1527A	GG/GC/CC	9/39/27	62.00%	64.80%
ABCA7	rs3764644	(19p13|NM_019112.3) c.183G>T, p.L61L	GG/GT/TT	64/11/0	7.33%	9.20%
ABCA7	rs3764645	(19p13|NM_019112.3) c.563A>G, p.E188G	AA/AG/GG	23/35/17	46.00%	43.60%
ABCA7	rs3764647	(19p13|NM_019112.3) c.1184A>G p.H395R	AA/AG/GG	64/11/0	7.33%	9.23%
ABCA7	rs3764648	(19p13|NM_019112.3) c.1215+10T>C	TT/TC/CC	7/36/32	66.67%	66.9%^
ABCA7	rs3764652	(19p13|NM_019112.3) c.3027C>T, p.A1009A	CC/CT/TT	30/34/11	37.33%	33.50%
ABCA7	rs4147912	(19p13|NM_019112.3) c.2380+8A>C	AA/AC/CC	0/18/57	88.00%	80.50%
ABCA7	rs4147914	(19p13|NM_019112.3) c.2385G>A, p.L795L	GG/GA/AA	26/39/10	39.33%	39.40%
ABCA7	rs4147915	(19p13|NM_019112.3) c.2421C>A, p.V807V	CC/CA/AA	28/37/10	38.00%	37.10%
ABCA7	rs4147918	(19p13|NM_019112.3) c.2380+8A>C	AA/AG/GG	59/16/0	10.67%	17.40%
ABCA7	rs4147920	(19p13|NM_019112) c.5571-7T>C	TT/TC/CC	59/16/0	10.67%	17.36%
ABCA7	rs4147921	(19p13|NM_019112.3) c.5592T>C, p.A1864A	TT/TC/CC	59/16/0	10.67%	15.30%
ABCA7	rs4147930	(19p13|NM_019112.3) c.5985G>A, p.L1995L	GG/GA/AA	21/37/17	47.33%	43.70%
ABCA7	rs4147934	(19p13|NM_019112.3) c.6133G>T, p.A2045S	GG/GT/TT	23/36/16	45.33%	43.30%
ABCA7	rs78320196	(19p13|NM_019112.3) c.5487T>C, p.N1829N	TT/TC/CC	69/6/0	4.00%	3.40%
ABCA7	rs881768	(19p13|NM_019112.3) c.4239A>G, p.R1413R	AA/AG/GG	30/34/11	37.33%	39.30%
BACE1	rs490460	(11q23|NM_001207048.1) c.540+5G>T	GG/GT/TT	0/10/65	93.33%	93.20%
BACE1	rs638405	(11q23|NM_012104.4) c.786G>C p.V262V	GG/GC/CC	17/32/26	56.00%	64.98%

BIN1	rs1060743	(2q14|NM_139343.2) c.486T>C p.T162T	TT/TC/CC	27/38/10	38.67%	45.34%
BIN1	rs1137845	(2q14|NM_139343.2) c.714C>T p.Y238Y	CC/CT/TT	54/20/1	14.67%	15.08%
BIN1	rs2228955	(2q14|NM_139343.2) c.894G>A p.S298S	GG/GA/AA	54/20/1	14.67%	15.87%
BIN1	rs2276579	(2q14|NM_139343.2) c.957C>T p.A319A	CC/CT/TT	69/6/0	4.00%	4.86%
BIN1	rs61748157	(2q14|NM_004305.3) c.682-4G>A	GG/GA/AA	72/3/0	2.00%	1.60%
BIN1	rs72481904	(2q14|NM_004305.3) c.605+10A>G	AA/AG/GG	29/37/9	36.67%	38.80%
CLU	rs7982	(8p21|NM_001831.3) c.789T>C p.H263H	TT/TC/CC	0/25/50	83.33%	78.87%
CR1	rs2274567	(1q32|NM_000573.3) c.3623A>G, p.H1208R	AA/AG/GG	54/19/2	15.33%	32.70%
CR1	rs2296160	(1q32|NM_000573.3) c.5905A>G, p.T1969A	AA/AG/GG	8/35/32	66.00%	68.90%
CR1	rs3737002	(1q32|NM_000573.3) c.4223C>T, p.T1408M	CC/CT/TT	19/44/12	45.33%	32.60%
CR1	rs3811381	(1q32|NM_000651.4) c.6830C>G p.P2277R	CC/CG/GG	54/19/2	15.33%	32.24%
CR1	rs4844600	(1q32|NM_000651.4) c.180A>G p.E60E	AA/AG/GG	9/41/25	60.67%	64.88%
CR1	rs6691117	(1q32|NM_000573.3) c.4843A>G, p.I1615V	AA/AG/GG	53/18/4	17.33%	34.30%
MS4A6A	rs12453	(11q12|NM_152851.2) c.327A>G p.L109L	AA/AG/GG	52/19/4	18.00%	22.52%
MS4A6A	rs7232	(11q12|NM_001330275.1) c.637A>T, p.T213S	AA/AT/TT	65/10/0	6.67%	5.80%
MS4A6E	rs2289613	(11q12|NM_139249.2) c.300T>C p.Y100Y	TT/TC/CC	57/16/2	13.33%	15.28%
MS4A6E	rs2304933	(11q12|NM_139249.2) c.139G>T p.V47F	GG/GT/TT	31/36/8	34.67%	25.50%
MS4A6E	rs2304934	(11q12|NM_139249.2) c.28A>G p.T10A	AA/AG/GG	31/36/8	34.67%	25.50%
MS4A6E	rs2304935	(11q12|NM_139249.2) c.16A>G p.I6V	AA/AG/GG	31/36/8	34.67%	25.40%
NME8	rs10250905	(7p15|NM_016616.4) c.622T>C, p.C208R	TT/TC/CC	19/34/22	52.00%	54.70%
NME8	rs1530822	(7p15|NM_016616.4) c.1140-10T>G	TT/TG/GG	6/27/42	74.00%	74.20%
NME8	rs2598044	(7p15|NM_016616.4) c.177C>T p.D59D	CC/CT/TT	53/19/3	16.67%	18.20%
NME8	rs2722372	(7p15|NM_016616.4) c.128G>A, p.R43K	GG/GA/AA	53/19/3	16.67%	18.20%
NME8	rs3213975	(7p15|NM_016616.4) c.1603T>C p.L535L	TT/TC/CC	38/32/5	28.00%	22.92%
NME8	rs41276027	(7p15|NM_016616.4) c.1479A>T p.I493I	AA/AT/TT	53/20/2	16.00%	13.59%
NME8	rs62001868	(7p15|NM_016616.4) c.840A>G p.R280R	AA/AG/GG	48/24/3	20.00%	22.22%
PICALM	rs592297	(11q14|NM_007166.3) c.522G>A p.Q174Q	GG/GA/AA	4/28/43	76.00%	68.15%
PICALM	rs694353	(11q14|NM_007166.3) c.1770T>G p.A590A	TT/TG/GG	4/32/39	73.33%	65.77%
PLAU	rs2227564	(10q24|NM_002658.4) c.422T>C, p.L141P	TT/TC/CC	12/31/32	63.33%	63.50%
PLAU	rs2227566	(10q24|NM_001145031.1) c.630-7C>T	CC/CT/TT	59/14/2	12.00%	12.30%
PLAU	rs2227568	(10q24|NM_002658.3) c.822C>T p.N274N	CC/CT/TT	37/31/7	30.00%	29.37%
SORL1	rs12364988	(11q23|NM_003105.5) c.807T>C p.H269H	TT/TC/CC	5/70/0	46.67%	31.15%
SORL1	rs1699102	(11q23|NM_003105.5) c.3738C>T p.N1246N	CC/CT/TT	64/11/0	7.33%	13.79%
SORL1	rs2070045	(11q23|NM_003105.5) c.3561T>G p.S1187S	TT/TG/GG	11/39/25	59.33%	56.85%
SORL1	rs2276412	(11q23|NM_003105.5) c.4176C>T p.N1392N	CC/CT/TT	70/5/0	3.33%	6.45%
SORL1	rs2298813	(11q23|NM_003105.5) c.1582G>A, p.A528T	GG/GA/AA	50/23/2	18.00%	12.10%
SORL1	rs3824968	(11q23|NM_003105.5) c.4752T>A p.A1584A	TT/TA/AA	11/36/28	61.33%	58.83%

[*]The actual alternate allele frequency from the AD cohort in this study.

[#]The alternate allele frequency from 1000 Genomes database.

[^]The alternate allele frequency from gnomAD database.

Supplementary Table 2. Effect of 69 SNPs on CSF Aβ$_{42}$, p-tau, and p-tau/Aβ$_{42}$

Gene	SNP	Cases	Aβ$_{42}$ (-/het/hom) (pg/ml)	p	P-tau (-/het/hom) (pg/ml)	p	P-tau/Aβ42 (-/het/hom)	p
APOE	rs429358	47/20/8	543.3±191.9/513.0±141.2/428.2±120.1	0.363*	74.1±35.8/68.4±24.3/87.1±20.4	0.242*	0.15±0.09/0.14±0.05/0.22±0.07	0.050*
APOE	rs7412	72/3/0	509.9±162.7/836.7±197.9	0.006#	75.3±31.7/42.5±13.9	0.079#	0.16±0.08/0.05±0.00	0.022#
A2M	rs11609582	71/4/0	523.7±176.8/509.0±161.5	0.776	75.6±31.8/44.7±16.6	0.029	0.16±0.08/0.11±0.09	0.12
A2M	rs1799759	71/4/0	523.7±176.8/509.0±161.5	0.776	75.6±31.8/44.7±16.6	0.029	0.16±0.08/0.11±0.09	0.12
A2M	rs669	63/12/0	518.3±164.9/547.4±228.0	0.751	72.8±27.0/80.1±51.7	0.522	0.15±0.07/0.17±0.12	0.377
ABCA7	rs3745842	30/34/11	551.5±212.1/497.1±154.4/525.2±113.0	0.592	88.0±37.5/65.9±25.9/60.7±14.1	0.025	0.18±0.09/0.15±0.07/0.12±0.04	0.035
ABCA7	rs3752232	64/11/0	535.2±175.5/452.0±161.3	0.186	74.6±32.4/70.2±29.8	0.633	0.15±0.07/0.17±0.10	0.517
ABCA7	rs3752233	58/17/0	524.8±163.3/516.6±215.9	0.833	72.4±28.6/79.5±41.6	0.387	0.15±0.07/0.18±0.10	0.239
ABCA7	rs3752234	2/26/47	796.0±150.2/539.6±232.8/502.1±124.5	0.061	57.5±38.2/69.3±39.0/77.3±27.2	0.546	0.07±0.03/0.15±0.10/0.16±0.06	0.469
ABCA7	rs3752237	0/19/56	568.4±255.9/507.6±137.2	0.242	75.3±42.2/73.5±27.9	0.834	0.16±0.11/0.15±0.07	0.839
ABCA7	rs3752239	60/15/0	520.4±162.3/533.3±225.2	0.847	73.5±29.2/75.8±42.1	0.782	0.15±0.07/0.16±0.10	0.793
ABCA7	rs3752240	59/13/3	536.5±172.2/428.1±117.9/667.6±307.3	0.007	75.0±33.3/74.4±27.8/52.0±6.0	0.503	0.16±0.08/0.18±0.06/0.09±0.03	0.191
ABCA7	rs3752241	56/19/0	527.9±176.6/508.4±173.9	0.797	72.0±28.9/80.0±39.5	0.385	0.16±0.07/0.17±0.10	0.252
ABCA7	rs3752243	27/35/13	548.5±209.5/504.9±160.7/518.4±134.8	0.748	87.9±39.4/68.8±25.8/59.3±15.8	0.074	0.18±0.09/0.15±0.07/0.12±0.06	0.068
ABCA7	rs3752246	9/39/27	672.4±205.1/498.7±150.7/508.3±179.4	0.047	93.0±39.2/78.8±33.7/60.7±20.3	0.011	0.15±0.08/0.17±0.09/0.13±0.06	0.135
ABCA7	rs3764644	64/11/0	535.2±175.5/452.0±161.3	0.186	74.6±32.4/70.2±29.8	0.633	0.15±0.07/0.17±0.10	0.517
ABCA7	rs3764645	23/35/17	585.3±204.9/483.4±151.1/520.1±162.5	0.23	82.7±44.2/73.5±26.1/63.2±17.8	0.113	0.16±0.11/0.16±0.06/0.13±0.06	0.36
ABCA7	rs3764647	64/11/0	535.2±175.5/452.0±161.3	0.186	74.6±32.4/70.2±29.8	0.633	0.15±0.07/0.17±0.10	0.517
ABCA7	rs3764648	7/36/32	626.6±173.3/509.4±158.5/515.5±190.4	0.337	81.2±45.7/84.3±33.2/60.8±21.4	0.005	0.14±0.08/0.18±0.08/0.13±0.06	0.03
ABCA7	rs3764652	30/34/11	551.4±212.1/497.1±154.4/525.2±113.0	0.592	88.0±37.5/65.9±25.9/60.7±14.1	0.025	0.18±0.09/0.15±0.07/0.12±0.04	0.035
ABCA7	rs4147912	0/18/57	573.4±262.3/507.0±136.0	0.218	76.0±43.4/73.4±27.7	0.745	0.16±0.11/0.15±0.07	0.732
ABCA7	rs4147914	26/39/10	508.7±186.6/515.4±178.4/589.6±122.1	0.618	60.9±21.6/83.0±31.8/72.6±44.0	0.018	0.13±0.06/0.18±0.08/0.13±0.10	0.085
ABCA7	rs4147915	28/37/10	552.1±206.6/501.6±146.1/520.7±183.8	0.617	78.2±42.5/73.1±25.0/65.5±17.1	0.544	0.16±0.10/0.16±0.06/0.14±0.06	0.806
ABCA7	rs4147918	59/16/0	531.7±175.4/490.8±175.2	0.39	71.9±28.3/81.9±42.7	0.274	0.15±0.07/0.18±0.10	0.065
ABCA7	rs4147920	59/16/0	531.7±175.4/490.8±175.2	0.39	71.9±28.3/81.9±42.7	0.274	0.15±0.07/0.18±0.10	0.065
ABCA7	rs4147921	59/16/0	531.7±175.4/490.8±175.2	0.39	71.9±28.3/81.9±42.7	0.274	0.15±0.07/0.18±0.10	0.065
ABCA7	rs4147930	21/37/17	557.3±202.7/499.2±165.5/507.7±152.4	0.237	98.7±39.1/65.9±23.9/61.0±19.1	<0.001	0.19±0.10/0.15±0.07/0.13±0.06	0.034

ABCA7	rs4147934	23/36/16	564.3±198.4/501.9±166.8/510.9±156.8	0.331	96.2±38.2/64.8±24.8/62.7±18.4	<0.001	0.19±0.09/0.14±0.07/0.13±0.06	0.055
ABCA7	rs78320196	69/6/0	516.5±168.8/596.9±241.8	0.279	74.4±32.5/69.8±25.0	0.783	0.16±0.08/0.15±0.10	0.667
ABCA7	rs881768	30/34/11	551.4±212.1/497.1±154.4/525.2±113.0	0.592	88.0±37.5/65.9±25.9/60.7±14.1	0.025	0.18±0.09/0.15±0.07/0.12±0.04	0.035
BACE1	rs490460	0/10/65	593.5±253.0/512.1±159.7	0.58	56.7±21.9/76.7±32.4	0.12	0.10±0.04/0.16±0.08	0.04
BACE1	rs638405	17/32/26	572.3±236.9/521.7±179.5/492.3±110.2	0.415	65.4±23.1/72.5±31.9/81.5±35.8	0.204	0.14±0.07/0.15±0.08/0.17±0.08	0.074
BIN1	rs1060743	27/38/10	461.7±140.8/560.3±183.4/546.6±196.3	0.205	81.0±27.4/71.1±35.3/65.9±28.1	0.375	0.19±0.07/0.14±0.08/0.13±0.07	0.09
BIN1	rs1137845	54/20/1	495.5±136.7/603.1±239.8/403.6	0.174	73.2±26.2/75.8±45.0/81.4	0.88	0.16±0.07/0.14±0.09/0.20	0.729
BIN1	rs2228955	54/20/1	495.5±136.7/603.1±239.8/403.6	0.174	73.2±26.2/75.8±45.0/81.4	0.88	0.16±0.07/0.14±0.09/0.20	0.729
BIN1	rs2276579	69/6/0	525.4±181.4/494.6±68.5	0.563	74.9±32.4/63.3±24.7	0.518	0.16±0.08/0.13±0.08	0.749
BIN1	rs61748157	72/3/0	528.2±176.5/397.1±46.1	0.244	73.4±32.3/89.2±7.9	0.419	0.15±0.08/0.23±0.01	0.193
BIN1	rs72481904	29/37/9	473.6±137.2/557.2±187.6/541.0±210.8	0.234	79.3±27.4/71.0±35.5/69.1±30.1	0.619	0.18±0.07/0.14±0.08/0.14±0.08	0.303
CLU	rs7982	0/25/50	560.1±207.6/504.4±155.2	0.327	87.7±43.2/67.2±21.8	0.006	0.17±0.10/0.15±0.06	0.077
CR1	rs2274567	54/19/2	527.3±186.7/489.2±95.2/725.4±389.2	0.102	74.9±34.5/71.2±24.5/76.7±28.6	0.95	0.16±0.08/0.15±0.07/0.14±0.11	0.915
CR1	rs2296160	8/35/32	487.1±121.2/536.7±187.2/516.9±175.4	0.609	81.7±18.2/75.8±41.9/70.0±19.7	0.664	0.18±0.06/0.16±0.09/0.15±0.07	0.879
CR1	rs3737002	19/44/12	495.0±164.1/543.5±181.8/492.0±169.4	0.64	76.6±24.3/75.1±37.0/65.9±20.4	0.541	0.17±0.08/0.15±0.07/0.16±0.09	0.844
CR1	rs3811381	54/19/2	527.3±186.7/489.2±95.2/725.4±389.2	0.102	74.9±34.5/71.2±24.5/76.7±28.6	0.95	0.16±0.08/0.15±0.07/0.14±0.11	0.915
CR1	rs4844600	9/41/25	457.0±128.0/551.4±186.4/500.1±165.9	0.247	81.0±18.0/74.7±39.7/70.4±18.8	0.764	0.19±0.06/0.15±0.08/0.16±0.07	0.69
CR1	rs6691117	53/18/4	526.0±188.2/499.3±99.9/589.1±275.0	0.528	74.8±34.9/72.8±24.8/68.1±19.8	0.945	0.16±0.08/0.15±0.07/0.13±0.07	0.856
MS4A6A	rs12453	52/19/4	518.8±171.7/550.3±196.1/446.8±102.6	0.499	73.8±32.1/72.6±34.4/82.5±17.5	0.72	0.16±0.08/0.15±0.09/0.19±0.05	0.538
MS4A6A	rs7232	65/10/0	517.5±167.2/558.3±226.8	0.264	75.0±32.7/67.7±25.9	0.225	0.16±0.08/0.15±0.08	0.468
MS4A6E	rs2289613	57/16/2	520.3±173.1/536.2±196.9/492.6±37.1	0.894	73.0±33.0/77.0±30.1/78.4±12.9	0.905	0.15±0.08/0.16±0.07/0.16±0.04	0.831
MS4A6E	rs2304933	31/36/8	546.1±166.2/511.3±197.3/485.4±76.9	0.451	68.5±25.5/78.1±37.7/76.7±24.4	0.413	0.14±0.06/0.17±0.09/0.16±0.06	0.125
MS4A6E	rs2304934	31/36/8	546.1±166.2/511.3±197.3/485.4±76.9	0.451	68.5±25.5/78.1±37.7/76.7±24.4	0.413	0.14±0.06/0.17±0.09/0.16±0.06	0.125
MS4A6E	rs2304935	31/36/8	546.1±166.2/511.3±197.3/485.4±76.9	0.451	68.5±25.5/78.1±37.7/76.7±24.4	0.413	0.14±0.06/0.17±0.09/0.16±0.06	0.125
NME8	rs10250905	19/34/22	500.5±139.8/543.6±179.8/510.4±197.6	0.709	77.2±33.8/73.5±38.3/72.0±16.4	0.958	0.17±0.08/0.15±0.08/0.16±0.07	0.745
NME8	rs1530822	6/27/42	655.0±290.4/548.3±174.3/487.8±146.6	0.042	76.8±24.8/74.8±36.1/73.0±30.4	0.903	0.15±0.09/0.15±0.08/0.16±0.08	0.852
NME8	rs2598044	53/19/3	531.1±174.2/514.4±190.3/433.7±58.3	0.447	75.9±31.1/71.1±33.5/58.8±41.7	0.746	0.16±0.07/0.16±0.09/0.14±0.12	0.604
NME8	rs2722372	53/19/3	531.1±174.2/514.4±190.3/433.7±58.3	0.447	75.9±31.1/71.1±33.5/58.8±41.7	0.746	0.16±0.07/0.16±0.09/0.14±0.12	0.604
NME8	rs3213975	38/32/5	525.3±183.0/501.9±156.3/639.9±216.2	0.428	68.4±21.4/83.4±40.4/55.9±21.4	0.064	0.15±0.07/0.18±0.08/0.10±0.06	0.065

NME8	rs41276027	53/20/2	517.9±175.0/540.1±184.1/484.3±140.6	0.829	71.4±28.8/80.3±40.1/79.8±16.5	0.548	0.15±0.07/0.16±0.09/0.17±0.01	0.861
NME8	rs62001868	48/24/3	528.6±192.7/486.0±117.1/727.4±151.4	0.078	74.8±34.7/72.0±27.7/77.3±16.5	0.806	0.16±0.09/0.15±0.06/0.11±0.03	0.558
PICALM	rs592297	4/28/43	502.3±73.2/539.5±156.6/514.1±193.7	0.89	79.7±45.9/79.8±36.6/69.7±27.1	0.417	0.16±0.08/0.16±0.08/0.15±0.08	0.944
PICALM	rs694353	4/32/39	502.3±73.2/547.9±174.2/504.6±183.2	0.77	79.7±45.9/74.9±37.5/72.6±25.5	0.886	0.16±0.08/0.15±0.08/0.16±0.08	0.869
PLAU	rs2227564	12/31/32	460.4±106.4/547.7±196.8/522.5±171.7	0.189	78.3±16.0/73.7±27.4/72.6±39.9	0.859	0.18±0.08/0.15±0.07/0.15±0.08	0.253
PLAU	rs2227566	59/14/2	521.8±188.4/518.3±123.4/590.7±12.9	0.825	75.3±32.4/65.9±29.2/92.8±39.0	0.39	0.16±0.08/0.13±0.06/0.16±0.06	0.578
PLAU	rs2227568	37/31/7	521.4±161.1/524.3±172.4/525.4±270.9	0.927	75.0±24.8/75.7±35.1/61.0±49.3	0.511	0.16±0.07/0.16±0.09/0.12±0.08	0.58
SORL1	rs12364988	5/70/0	380.8±129.8/533.1±174.1	0.096	63.2±33.3/74.8±31.8	0.286	0.17±0.07/0.16±0.08	0.703
SORL1	rs1699102	64/11/0	514.0±176.4/574.9±164.5	0.344	72.3±28.7/83.9±46.6	0.181	0.16±0.07/0.16±0.10	0.717
SORL1	rs2070045	11/39/25	580.1±172.2/491.9±151.3/546.2±205.4	0.281	70.8±22.4/78.3±36.8/68.7±26.7	0.261	0.13±0.06/0.17±0.09/0.14±0.07	0.099
SORL1	rs2276412	70/5/0	516.0±169.5/620.2±242.0	0.334	74.5±32.6/66.2±18.4	0.735	0.16±0.08/0.13±0.07	0.628
SORL1	rs2298813	50/23/2	533.2±159.4/507.7±211.8/442.1±101.3	0.704	73.0±35.3/75.7±25.0/79.7±14.1	0.959	0.15±0.08/0.17±0.08/0.19±0.08	0.519
SORL1	rs3824968	11/36/28	611.9±173.9/490.6±149.1/529.7±198.1	0.197	72.3±21.7/77.5±38.5/70.1±25.6	0.302	0.13±0.06/0.17±0.09/0.15±0.06	0.094

CSF data were compared by analysis of covariate (ANCOVA). Sex, age, disease course, and *APOE* status (ε4 carrier or non-carrier) were included in the model as fixed factor or covariate.[*]Sex, age, disease course, and rs7412 were included in the model as fixed factor or covariate. [#]Sex, age, disease course, and rs429358 were included in the model as fixed factor or covariate. -/het/hom, reference allele homozygote/heterozygote/alternate allele homozygote. Unadjusted means were displayed.

Supplementary Table 3. Effect of 69 SNPs on CSF t-tau and t-tau/Aβ$_{42}$

Gene	SNP	T-tau (-/het/hom) (pg/ml)	p	T-tau/Aβ42 (-/het/hom)	p
APOE	rs429358	707.2±862.9/621.8±493.1/746.8±366.0	0.514*	1.39±1.42/1.22±1.01/1.72±0.72	0.380*
APOE	rs7412	700.6±744.9/402.4±380.0	0.313#	1.42±1.27/0.56±0.64	0.165#
A2M	rs11609582	702.3±750.9/447.0±272.7	0.426	1.41±1.29/0.94±0.54	0.425
A2M	rs1799759	702.3±750.9/447.0±272.7	0.426	1.41±1.29/0.94±0.54	0.425
A2M	rs669	686.8±790.9/698.3±324.4	0.911	1.37±1.33/1.46±0.81	0.887
ABCA7	rs3745842	828.2±1006.8/616.0±469.7/532.6±472.5	0.515	1.57±1.59/1.30±0.95/1.12±1.09	0.621
ABCA7	rs3752232	725.0±782.1/477.5±287.6	0.36	1.43±1.33/1.09±0.66	0.462
ABCA7	rs3752233	704.1±791.9/636.1±508.0	0.862	1.39±1.30/1.36±1.13	0.972
ABCA7	rs3752234	346.1±282.2/577.7±506.6/764.6±842.1	0.564	0.41±0.28/1.25±1.14/1.50±1.33	0.454
ABCA7	rs3752237	608.1±487.4/716.0±803.4	0.656	1.26±1.11/1.42±1.31	0.675
ABCA7	rs3752239	711.4±780.3/597.6±525.1	0.629	1.42±1.30/1.23±1.13	0.636
ABCA7	rs3752240	691.8±805.1/703.8±403.9/562.3±407.1	0.897	1.37±1.37/1.54±0.70/0.92±0.68	0.709
ABCA7	rs3752241	696.9±801.1/664.4±507.3	0.963	1.38±1.32/1.39±1.09	0.869
ABCA7	rs3752243	838.9±1060.1/648.2±457.5/485.7±447.4	0.47	1.58±1.67/1.35±0.94/1.05±1.03	0.561
ABCA7	rs3752246	1123.7±1763.7/680.7±424.0/555.1±481.9	0.174	1.83±2.76/1.41±0.78/1.19±1.08	0.449
ABCA7	rs3764644	725.0±782.1/477.5±287.6	0.36	1.43±1.33/1.09±0.66	0.462
ABCA7	rs3764645	769.0±1145.3/734.3±513.9/486.0±271.7	0.382	1.41±1.81/1.54±1.03/1.01±0.60	0.331
ABCA7	rs3764647	725.0±782.1/477.5±287.6	0.36	1.43±1.33/1.09±0.66	0.462
ABCA7	rs3764648	1357.6±1977.5/647.7±428.1/588.4±459.5	0.079	2.22±3.07/1.34±0.81/1.25±1.02	0.252
ABCA7	rs3764652	828.2±1006.8/616.0±469.7/532.6±472.5	0.515	1.57±1.59/1.30±0.95/1.12±1.09	0.621
ABCA7	rs4147912	626.0±495.0/708.5±798.2	0.746	1.30±1.13/1.41±1.30	0.784
ABCA7	rs4147914	553.6±424.4/694.1±449.4/1018.5±1706.2	0.299	1.18±0.96/1.43±0.88/1.72±2.65	0.523
ABCA7	rs4147915	711.5±1048.4/703.7±508.2/569.2±298.1	0.861	1.34±1.66/1.46±1.03/1.19±0.68	0.811
ABCA7	rs4147918	691.4±784.3/678.5±532.8	0.991	1.37±1.30/1.44±1.13	0.82
ABCA7	rs4147920	691.4±784.3/678.5±532.8	0.991	1.37±1.30/1.44±1.13	0.82
ABCA7	rs4147921	691.4±784.3/678.5±532.8	0.991	1.37±1.30/1.44±1.13	0.82
ABCA7	rs4147930	874.0±1175.6/645.4±487.3/553.9±407.2	0.361	1.61±1.84/1.35±0.98/1.17±0.91	0.567
ABCA7	rs4147934	812.9±1139.3/703.5±514.5/476.9±263.0	0.336	1.50±1.79/1.47±1.05/1.00±0.59	0.381
ABCA7	rs78320196	714.6±759.0/389.9±193.7	0.395	1.43±1.29/0.80±0.55	0.292
ABCA7	rs881768	828.2±1006.8/616.0±469.7/532.6±472.5	0.515	1.57±1.59/1.30±0.95/1.12±1.09	0.621
BACE1	rs490460	493.2±579.2/718.7±755.0	0.419	0.93±1.27/1.45±1.25	0.252
BACE1	rs638405	582.6±499.6/714.0±1012.2/726.8±396.7	0.664	1.21±1.12/1.38±1.63/1.49±0.75	0.717
BIN1	rs1060743	616.3±436.1/751.1±943.2/646.9±443.2	0.785	1.35±0.90/1.43±1.56/1.28±0.84	0.882
BIN1	rs1137845	636.5±448.4/807.6±1228.2/1125.6	0.622	1.33±0.92/1.45±1.93/2.79	0.431
BIN1	rs2228955	636.5±448.4/807.6±1228.2/1125.6	0.622	1.33±0.92/1.45±1.93/2.79	0.431
BIN1	rs2276579	675.1±750.2/844.3±550.0	0.656	1.35±1.27/1.77±1.21	0.479
BIN1	rs61748157	692.6±748.6/593.9±250.3	0.849	1.38±1.28/1.46±0.43	0.893
BIN1	rs72481904	650.2±444.7/721.5±953.2/677.7±448.8	0.898	1.38±0.90/1.39±1.57/1.34±0.82	0.837

CLU	rs7982	800.0±1085.2/633.2±478.4	0.456	1.50±1.70/1.32±0.98	0.628
CR1	rs2274567	663.8±811.9/765.4±512.3/632.2±352.6	0.815	1.31±1.35/1.60±1.02/1.17±1.11	0.633
CR1	rs2296160	718.8±440.2/682.4±942.0/688.0±520.7	0.985	1.46±0.66/1.31±1.51/1.44±1.09	0.868
CR1	rs3737002	629.7±344.7/780.4±915.8/445.7±232.1	0.472	1.37±0.70/1.49±1.55/1.00±0.52	0.574
CR1	rs3811381	663.8±811.9/765.4±512.3/632.2±352.6	0.815	1.31±1.35/1.60±1.02/1.17±1.11	0.633
CR1	rs4844600	624.6±389.9/690.3±889.0/709.1±542.5	0.881	1.38±0.62/1.29±1.44/1.52±1.14	0.732
CR1	rs6691117	671.0±818.0/765.1±525.1/579.6±311.0	0.78	1.33±1.35/1.59±1.06/1.15±0.79	0.629
MS4A6A	rs12453	609.7±387.9/852.4±1280.5/937.9±745.6	0.444	1.26±0.83/1.59±2.05/1.92±1.17	0.469
MS4A6A	rs7232	676.5±748.3/768.0±669.4	0.706	1.36±1.26/1.55±1.33	0.663
MS4A6E	rs2289613	618.2±427.5/935.9±1371.3/720.0±486.7	0.462	1.29±0.92/1.70±2.10/1.50±1.10	0.604
MS4A6E	rs2304933	559.3±410.6/752.2±982.1/904.2±113.4	0.468	1.10±0.82/1.51±1.61/1.91±0.46	0.223
MS4A6E	rs2304934	559.3±410.6/752.2±982.1/904.2±113.4	0.468	1.10±0.82/1.51±1.61/1.91±0.46	0.223
MS4A6E	rs2304935	559.3±410.6/752.2±982.1/904.2±113.4	0.468	1.10±0.82/1.51±1.61/1.91±0.46	0.223
NME8	rs10250905	863.8±1230.4/663.2±525.0/576.9±359.5	0.418	1.67±1.88/1.29±1.05/1.28±0.85	0.477
NME8	rs1530822	669.8±377.5/867.1±1071.2/576.7±441.8	0.22	1.28±0.81/1.65±1.74/1.22±0.89	0.322
NME8	rs2598044	392.2±53.9/1306.5±299.7/160.5±92.7	0.332	1.27±0.85/1.72±2.06/1.28±0.42	0.393
NME8	rs2722372	392.2±53.9/1306.5±299.7/160.5±92.7	0.332	1.27±0.85/1.72±2.06/1.28±0.42	0.393
NME8	rs3213975	576.9±457.4/811.0±999.7/755.3±244.5	0.485	1.22±0.96/1.58±1.60/1.32±0.58	0.529
NME8	rs41276027	657.3±800.5/731.6±504.0/1089.5±1177.1	0.593	1.30±1.31/1.53±1.10/1.98±1.86	0.59
NME8	rs62001868	692.9±848.7/700.0±508.5/529.7±170.5	0.85	1.40±1.41/1.42±0.98/0.75±0.30	0.585
PICALM	rs592297	683.2±480.2/803.5±1025.3/614.4±494.3	0.607	1.33±0.86/1.54±1.65/1.28±0.98	0.739
PICALM	rs694353	683.2±480.2/759.0±968.0/631.5±510.7	0.819	1.33±0.86/1.45±1.57/1.33±1.01	0.954
PLAU	rs2227564	728.9±509.8/702.0±452.4/660.6±998.2	0.896	1.62±1.07/1.41±0.89/1.26±1.60	0.72
PLAU	rs2227566	733.8±812.3/497.2±267.0/698.1±333.2	0.42	1.48±1.38/1.00±0.57/1.18±0.54	0.364
PLAU	rs2227568	671.9±451.2/617.5±438.9/1092.2±2062.5	0.463	1.39±0.95/1.26±0.87/1.87±3.16	0.657
SORL1	rs12364988	900.5±647./673.5±742.3	0.538	2.06±1.32/1.33±1.25	0.225
SORL1	rs1699102	697.7±782.1/636.4±369.4	0.858	1.41±1.33/1.20±0.75	0.655
SORL1	rs2070045	712.1±533.0/759.8±939.7/567.4±351.9	0.508	1.30±0.93/1.56±1.55/1.15±0.77	0.384
SORL1	rs2276412	701.1±754.8/515.4±343.3	0.535	1.41±1.29/0.94±0.63	0.403
SORL1	rs2298813	771.1±852.9/536.3±382.6/379.1±65.1	0.314	1.51±1.43/1.15±0.80/0.90±0.35	0.402
SORL1	rs3824968	708.5±536.6/792.6±969.6/547.3±344.5	0.36	1.27±0.96/1.61±1.59/1.13±0.75	0.265

CSF data were compared by analysis of covariate (ANCOVA). Sex, age, disease course, and *APOE* status (ε4 carrier or non-carrier) were included in the model as fixed factor or covariate. *Sex, age, disease course, and rs7412 were included in the model as fixed factor or covariate. #Sex, age, disease course, and rs429358 were included in the model as fixed factor or covariate. -/het/hom, reference allele homozygote/heterozygote/alternate allele homozygote. Unadjusted means were displayed.
